# Phenotypic and Genetic Evolutions of a Porcine Reproductive and Respiratory Syndrome Modified Live Vaccine after Limited Passages in Pigs

**DOI:** 10.3390/vaccines9040392

**Published:** 2021-04-16

**Authors:** Julie Eclercy, Patricia Renson, Edouard Hirchaud, Mathieu Andraud, Véronique Beven, Frédéric Paboeuf, Nicolas Rose, Yannick Blanchard, Olivier Bourry

**Affiliations:** Agence Nationale de Sécurité Sanitaire de l’Alimentation, de l’Environnement et du Travail (Anses), Laboratoire de Ploufragan-Plouzané-Niort, BP 53, 22440 Ploufragan, France; julie.eclercy@gmail.com (J.E.); patricia.renson@anses.fr (P.R.); edouard.hirchaud@anses.fr (E.H.); mathieu.andraud@anses.fr (M.A.); veronique.beven@anses.fr (V.B.); frederic.paboeuf@anses.fr (F.P.); nicolas.rose@anses.fr (N.R.); yannick.blanchard@anses.fr (Y.B.)

**Keywords:** PRRS virus, modified live vaccine, attenuation, variants, mutation

## Abstract

Modified live vaccines (MLVs) against the porcine reproductive and respiratory syndrome virus (PRRSV) have been regularly associated with safety issues, such as reversion to virulence. In order to characterize the phenotypic and genetic evolution of the PRRSV-1 DV strain from the Porcilis^®^ PRRS MLV after limited passages in pigs, three in vivo experiments were performed. Trial#1 aimed (i) at studying transmission of the vaccine strain from vaccinated to unvaccinated contact pigs. Trial#2 and Trial#3 were designed (ii) to assess the reproducibility of Trial#1, using another vaccine batch, and (iii) to compare the virulence levels of two DV strains isolated from vaccinated (passage one) and diseased contact pigs (passage two) from Trial#1. DV strain isolates from vaccinated and contact pigs from Trial#1 and Trial#2 were submitted to Next-Generation Sequencing (NGS) full-genome sequencing. All contact animals from Trial#1 were infected and showed significantly increased viremia compared to vaccinated pigs, whereas no such change was observed during Trial#2. In Trial#3, viremia and transmission were higher for inoculated pigs with passage two of the DV strain, compared with passage one. In this study, we showed that the re-adaptation of the DV strain to pigs is associated with faster replication and increased transmission of the vaccine strain. Punctually, a decrease of attenuation of the DV vaccine strain associated with clinical signs and increased viremia may occur after limited passages in pigs. Furthermore, we identified three mutations linked to pig re-adaptation and five other mutations as potential virulence determinants.

## 1. Introduction

The porcine reproductive and respiratory syndrome virus (PRRSV) is one of the most important swine diseases in the world. It induces an enormous economic burden due to reproductive failure in sows and a complex respiratory syndrome in pigs of all ages [1,2]. PRRSV belong to the Arteriviridae family [3], genus *Betaarterivirus*, with two species: *Betaarterivirus suid 1*, formerly PRRSV-1, which has a European origin; and PRRSV-2, renamed *Betaarterivirus suid 2*, originating from North America. Both genera share ~60% genetic identity at the nucleotide level [4]. The PRRSV single-stranded RNA (+) genome possesses at least 10 open reading frames (ORFs) flanked by a 5′ leader and 3′ untranslated region followed by a poly-A tail. The non-structural proteins (NSPs), encoded by ORF1a and ORF1b, have protease, replicase and host gene modulation functions [5]. The 3′ end of the genome codes for at least eight structural proteins; three major proteins, namely glycoprotein GP5, matrix (M) and nucleocapsid (N) are encoded by ORF5, ORF6 and ORF7, respectively; and five minor proteins, namely GP2, GP3 and GP4, which compose surface glycoproteins and are derived from ORF2, ORF3 and ORF4, respectively, and two small non-glycosylated 2b and 5a proteins translated from ORF2b and ORF5a, respectively [6]. PRRSV displays a high mutation rate due to the infidelity of the viral RNA-dependent RNA polymerase during replication [7]. Recombination is also a major mechanism contributing with mutations to the emergence and the evolution of new PRRSV variants [6].

Since the discovery of PRRSV, several modified live vaccines (MLVs) have been developed after attenuation of wild-type strains during multiple in vitro passages in cell lines such as MA-104 or MARC-145 [8]. MLVs have been licensed in various countries, depending on circulating viral species, against PRRSV-1 or PRRSV-2, named MLV1 and MLV2, respectively, and still represent the major prophylactic tool used against PRRSV [9].

Attenuated PRRSV vaccine strains are characterized by the same great genetic instability as wild-type strains. Both mutations and recombination phenomenon can lead to virulence recovery [10]. The return to virulence (reversion) by mutations of the vaccine strains can cause clinical diseases, both reproductive and respiratory, and affect growth performance [11,12,13]. MLV reversion is a gradual process starting with the decrease of the attenuated phenotype of the vaccine strain. Several studies aimed at identifying potential mutations associated with attenuation and reversion of PRRSV vaccine strains [14,15,16]. However, all of them focused on MLV2, and to the best of our knowledge, no studies explored the loss of attenuation and reversion of MLV1 strains.

In this context, the objective of the present study was to monitor the phenotypic and genetic changes of a PRRSV MLV1 (Porcilis^®^ PRRS vaccine, DV strain) during two consecutive passages in pigs. To fulfill this objective, we used a study design relying on three in vivo studies combined with extensive genetic analysis of the DV vaccine strains. In a first trial (Trial#1) transmission of the DV strain from vaccinated to unvaccinated contact pigs was assessed, with changes in phenotype of the vaccine strain observed in contact animals. In Trial#2, we assessed the reproducibility of Trial#1 with another batch of the Porcilis^®^ PRRS vaccine. At last, in Trial#3, we compared the virulence from the strain isolates from vaccinated and unvaccinated contact pigs issued from Trial#1, to confirm the modification of phenotype observed during the transmission assay. To complement our study, we isolated and sequenced the DV strain isolates from each vaccinated and unvaccinated contact pigs from Trial#1 and Trial#2 to monitor the genetic evolution of the DV vaccine strain at the full-genome level during the two consecutive passages in pigs.

## 2. Materials and Methods

### 2.1. Animal Experiments

#### 2.1.1. Viruses

For Trial#1 and Trial#2, two different batches of Porcilis^®^ PRRS vaccine (MSD, Beaucouzé, France; DV strain, GenBank accession No. KF991509.2) were used as recommended by the manufacturers: batch No. A142DB01 (P0-2012 strain) for Trial#1 (performed in 2012) and batch No. A207CB01 (P0-2017 strain) for Trial#2 (performed in 2017).

For Trial#3, the P1-I-2012 strain was isolated from heparinized blood at PRRSV viremia peak of a vaccinated piglet from Trial#1, and the P2-C-2012 strain from heparinized blood at PRRSV viremia peak of a contact piglet from the same Trial#1 presenting hyperthermia and higher PRRSV load in blood than vaccinated piglets. Both DV-like strains were isolated and then propagated in porcine alveolar macrophages (PAMs) as previously described [8]. The PAMs were obtained by bronchoalveolar lavages from lungs of specific pathogen free (SPF) piglets euthanized at 6–8 weeks old at Anses, Ploufragan-Plouzané-Niort laboratory. Cells harvest method are similar to procedures described previously [17,18].

Both Porcilis^®^ PRRS vaccine lyophilizates used in Trial#1 and Trial#2 were resuspended in the diluent provided with the vaccines as recommended by the manufacturers, and both P1-I-2012 and P2-C-2012 strain inocula were diluted in MEM (Corning, New York, NY, USA) culture medium. Equivalent viral titers determined in MARC-145 cells were used for inoculations: 10^4.2^ tissue culture infective dose 50 per milliliter (TCID_50_/mL) for the P0-2012 strain (from Porcilis^®^ PRRS vaccine batch No. A142DB01; Trial#1), 10^4.8^ TCID_50_/mL for the P0-2017 strain (from Porcilis^®^ PRRS vaccine batch No. A207CB01; Trial#2) and 10^4.5^ TCID_50_/mL for both P1-I-2012 and P2-C-2012 isolates. The MARC-145 cells were kindly provided by Pr. Moennig from the Federal Research Centre for Virus Diseases of Animals, Tubingen, Germany.

#### 2.1.2. Animal Experiments

**Figure 1:**
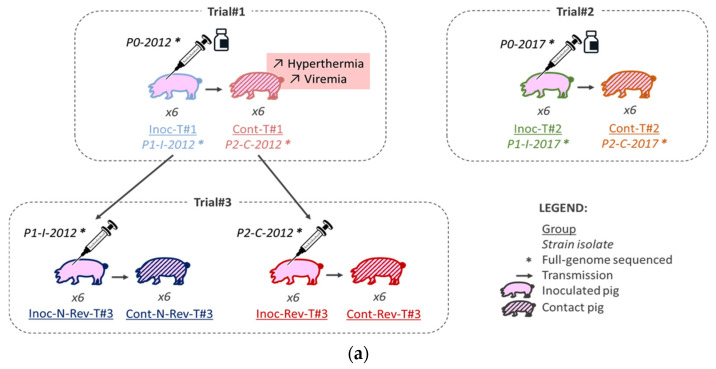

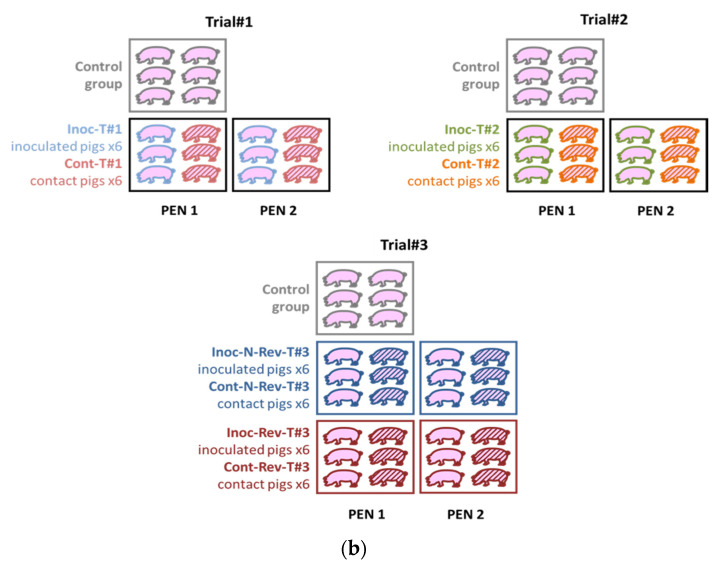
Global framework (**a**) and schemes (**b**) of the three different animal experiments.

The trials were performed in 2012 (Trial#1) and in 2017 (Trial#2 and Trial#3) in our Biosafety level 3 (BSL-3) animal facilities in Anses, Ploufragan-Plouzané-Niort laboratory, following the same experimental conditions (Figure 1a).

For each animal trial, one control group was included and all piglets were randomly stratified by gender, weight and litter in the different groups. In Trial#1 and Trial#2, 6 SPF Large White piglets were allocated to two different pens (separated by a solid plastic partition preventing direct contacts between pigs from different pens). In each pen, 3 of them were vaccinated intramuscularly (IM) in the neck with 2 mL of the Porcilis^®^ PRRS vaccine (MSD, Beaucouzé, France) corresponding to the inoculated Trial #1 (Inoc-T#1) and Inoc-T#2 vaccinated pigs, respectively (Figure 1a,b and Table 1). In Trial#3, 6 piglets in each group (3 per pen) were inoculated IM in the neck with 2 mL of the non-revertant (N-Rev) P1-I-2012 isolate composing the Inoc-N-Rev-T#3 inoculated group or with the revertant (Rev) P2-C-2012 isolate representing the Inoc-Rev-T#3 inoculated group (Figure 1a,b and Table 1). In all trials, 24 h after inoculation, 6 non-inoculated contact piglets (3 per pen) were added to each inoculated groups to evaluate viral transmission (Figure 1b and Table 1). The animals were monitored for 35 days post-inoculation (dpi), daily for rectal temperatures and food intake and weekly for body weight. Rectal temperatures over 40.0 °C were considered as hyperthermia. Limit points were determined in agreement with the ethical committee to respect animal welfare. Blood samples were collected from all pigs and nasal swabs only from inoculated and control animals before inoculation, and then twice a week until 5 weeks after inoculation. Blood for Trial#1, sera for Trial#2 and Trial#3 and nasal swabs suspended in RNAlater™ (Qiagen, Hilden, Germany) were stored at −80 °C until tested. All pigs were euthanized at 34–36 dpi, after anesthesia (Zoletil^®^, Virbac, Carros, France, using 15 mg/kg), by bleeding, and then necropsied. One N-Rev-T#3 contact pig from Trial#3 was euthanized before the end of experiment at 10 dpi independently of PRRSV infection (ethical euthanasia after crossing limit points due to generalized infection caused by non-typeable *Escherichia coli*).

#### 2.1.3. Ethical Statement

Trial#1’s protocol was approved on 11 December 2012, by the Ethics Committee, number 16 under reference 12-063, approval No. 11/12/12-6. Trial#2 and Trial#3 protocols were approved on 13 December 2016, by the Ethics Committee, number 16 under reference 16-088, and were also agreed by the French Ministry of Research, under reference 7788-201611281003891_v1.

#### 2.1.4. Quantification of PRRS Viral Genome Load

For Trial#1 samples, as previously described [19], viral RNA was extracted from EDTA-stabilized blood, using the RNeasy mini kit (Qiagen, Hilden, Germany) according to the manufacturer’s instructions, whereas for Trial#2 and Trial#3 samples, viral RNA was extracted from serum or nasal swab supernatant, using the NucleoSpin^®^ RNA 8 virus kit (Macherey-Nagel, Düren, Germany) according to the manufacturer’s instructions. For the 3 different trials, PRRSV ORF7 and porcine β-actine gene expression were quantified by using the same in-house duplex qRT-PCR [19].

For Trial#2 and Trial#3, and for RNA extracted from serum, the genomic viral load was quantified by using a standard viral range of the Porcilis^®^ PRRS DV strain (with a known virus titer obtained in MARC-145 cells) diluted in serum from SPF pigs. Results were expressed as equivalent (eq) TCID_50_/mL of serum, as previously described [20]. For RNA extracted from blood of Trial#1 and from nasal-swab supernatants of Trial#3, viral load and virus shedding were quantified by relative quantification, using the ΔΔCt method, and results were expressed in Log2 R (relative amount R =  2^−ΔΔC^), as previously described [19].

Control pigs remained PRRSV negative in serum, blood and nasal swabs throughout the study.

#### 2.1.5. Statistical Analyses

Data were analyzed with R software (v.3.2.1). Due to the limited number of animals in each group of our 3 trials, we were not able to observe a normal distribution for our data. For this main reason, we used non-parametric tests for the statistical analyses. Rectal temperatures and average daily weight gains (ADWG) were compared between the different groups for each measured point, using the non-parametric Kruskal–Wallis test. Then, a Wilcoxon pairwise test with the Holm’s method for adjustment of multiple comparisons was used for group comparison. The genomic viral loads in blood samples and in nasal swabs were evaluated by calculating the area under the curve (AUC) for each pig profile. A Kruskal–Wallis test followed by the Wilcoxon pairwise test was then used to assess differences between groups. Differences in viral loads between inoculated and contact pigs from both Trial#1 and Trial#2 were analyzed only at viremia peak for all animals with a Kruskal–Wallis test and a Wilcoxon pairwise test. Results with *p*-values ≤ 0.05 were considered statistically significant. Data obtained from the N-Rev-T#3 contact pig from Trial#3 euthanized at 10 dpi were excluded from the averages of rectal temperature and from the mean viremia AUC.

#### 2.1.6. Transmission Parameters 

Longitudinal data from PRRSV genome detection in sera of inoculated and unvaccinated contact animals from the 2 different groups of Trial#3 were used to estimate transmission parameters by a Bayesian inference, as previously described [21]. Two parameters were determined: the latency (representing the time lapse when an infected pig becomes infectious) expressed in days, and the daily transmission rate or number of infected pigs by one infectious pig per day. Statistical analyses to compare transmission parameters between both groups were made by using the Bayesian Estimation Supersedes *t*-Test package.

### 2.2. Genetic Characterization

#### 2.2.1. Strains

In order to characterize the genetic evolution of the DV strain after 2 in vivo passages, several isolates presenting different number of passages in pigs were isolated in PAMs from serum or heparinized blood at viremia peak, sequenced and genetically characterized (Figure 1a).

Vaccine strains from both Porcilis^®^ PRRS batches used in Trial#1 and Trial#2 represented the in vivo passage number 0 (P0), respectively, named P0-2012 and P0-2017 (Figure 1a and Table 1). Vaccine strains isolated from the 12 vaccinated animals from Trial#1 and Trial#2 represented the in vivo passage number 1 (P1), respectively, named P1-I-2012 and P1-I-2017 (Figure 1a). Finally, vaccine strains isolated from the 12 contact pigs from Trial#1 and Trial#2 represented the in vivo passage number 2 (P2), respectively named P2-C-2012 and P2-C-2017 (Figure 1a). In order to improve the likelihood of virus isolation, the vaccine strains were isolated from serum/blood samples of pigs at viremia peak for each animal (Table S1). Twenty-one isolates from P1 and P2 have been amplified in PAMs and 2 isolates (sequences from pigs No. 2012_4 and No. 2017_12) that did not grow in PAMs, were isolated and propaged in MARC-145 cells. One strain (P2-C-2012) could not be isolated either in PAMs or in MARC-145 cells. In total 25 vaccine isolates, from passage 0 to 2, were genetically characterized.

#### 2.2.2. Full-Genome Sequencing

All 25 isolates were concentrated using Amicon^®^ Ultra-15 100 K centrifugal filter devices (Merck, Darmstadt, Germany) and RNA was extracted using a standard Trizol LS reagent extraction protocol (Thermo Fisher Scientific, Waltham, MA, USA). Then, all the samples were sent to the Anses Next-Generation Sequencing (NGS) platform for full-genome sequencing by the RNAseq method, using Ion Torrent Proton technology as previously described [22]. An alignment on reference KF991509.2 of Porcilis^®^ PRRS DV strain was performed with bwa and the result of this alignment was used for an estimation (%) of the distribution of the different forms of PRRSV genomes present in both Porcilis^®^ PRRS vaccine batches and in the sample of each pig by counting the reads corresponding to the full-length sequence or the different variants described around the deleted zones.

All the full-genome sequences listed in this article are gathered into the BioProject No. PRJNA705101. The BioSample No. SAMN18137716 including the SRA No. SRR13855296 contains the 4 different PRRSV forms identified in both Porcilis^®^ PRRS vaccine batches including the DV FULL-LENGTH, LONG-DEL, SHORT-DEL and SHIFT-DEL full-genome sequences, respectively, deposited into GenBank under the accession Nos. MW674755, MW674756, MW674757 and MW674758.

#### 2.2.3. Genomic Analysis

Comparison of both full-genome sequences P0-2012 and P0-2017 from the two vaccine batches with the official reference sequence (at the time of the study) of the DV strain of the Porcilis^®^ PRRS deposited in GenBank under the accession No. KF991509.2 by MSD (Kenilworth, NJ, USA), was performed with MEGA7 software (Version 7.0.26) to identify deletions and mutations. Comparison of full-genome sequences between P0 (P0-2012; P0-2017), P1 (P1-I-2012; P1-I-2017) and P2 (P2-C-2012; P2-C-2017) was performed to identify non-conservative mutations between the different passages in pigs. Identity rates were determined after an alignment using the Multiple Sequence Comparison by Log-Expectation (MUSCLE) algorithm. Coverage rates of the two full-genome sequences from pig No. 2012_9 (short-deleted variant) and pig No. 2012_3 (shift-deleted variant) were sufficient to determine the deleted nature of variants but not enough for identification of single-nucleotide polymorphism (SNP) mutations, thus both sequences were excluded from the identification of mutations.

## 3. Results

### 3.1. Phenotypic Characterization

#### 3.1.1. Trial#1

**Figure 2:**
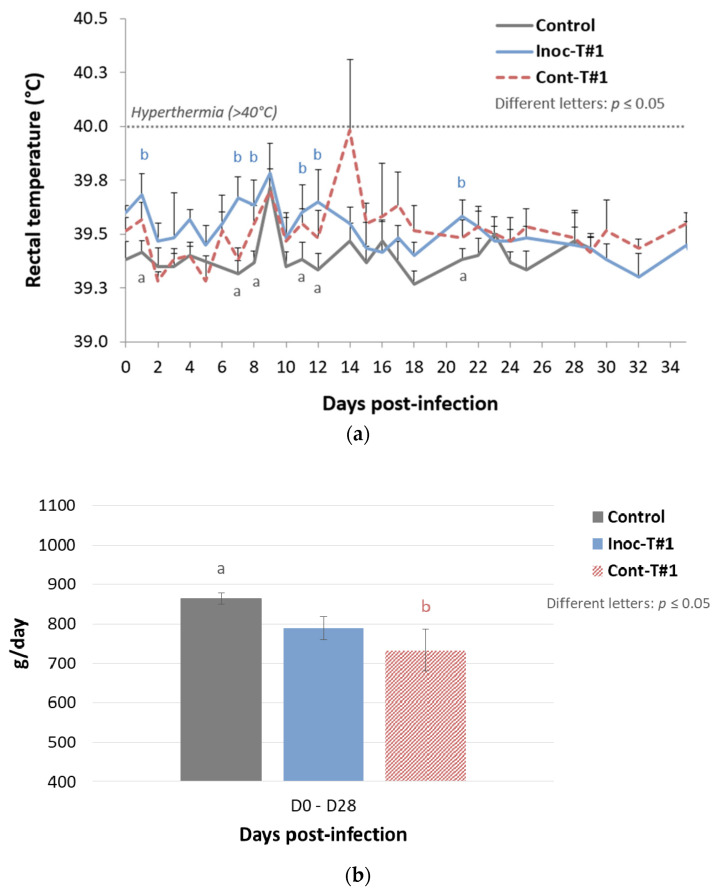

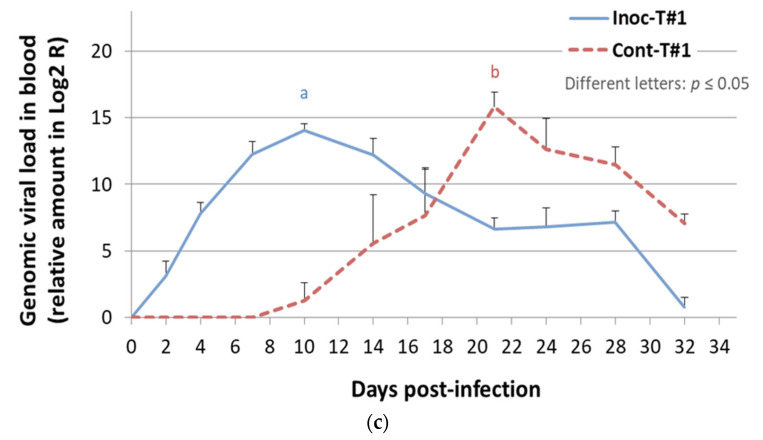
Results from Trial#1. Evolution of the mean rectal temperatures (°C) (**a**) and of the average daily weight gain (ADWG) (g/day) (**b**) in inoculated Trial#1 (Inoc-T#1) pigs and in contact Trial#1 (Cont-T#1) pigs after inoculation (day 0). Different letters (“a” and “b”) indicate that the groups are significantly different from each other with *p* ≤ 0.05. (**c**) Evolution of the mean genomic viral loads in blood (relative amount expressed in Log2 R) in Inoc-T#1 inoculated pigs and in Cont-T#1 contact pigs after inoculation (day 0). Different letters (“a” and “b”) indicate that the groups are significantly different at peak values from each other with *p* ≤ 0.05.

In this first trial, we aimed to assess the transmission of the DV vaccine strain (from vaccinated (inoculated) to unvaccinated contact pigs) and the possible phenotypic evolution of this strain after natural transmission. Starting with the evolution of rectal temperatures after inoculation: 1/6 inoculated pig and 3/6 contact pigs displayed hyperthermia (>40 °C) during the study, with a total of 4 days of hyperthermia detected during the follow-up period for the contact pigs and 1 day for the inoculated ones (Figure 2a). Regarding growth performances, a significant decrease of average daily weight gain (ADWG) was noticed for unvaccinated contact pigs from D0 to D28 pi, compared to the control group, whereas no significant difference was observed between vaccinated and control animals during the same period (Figure 2b).

Following with PRRSV genome quantification in blood samples, inoculated pigs showed a genomic viral load peak at 10 dpi with a relative amount of 14.03 ± 1.30 (Log2 R). However, contact pigs displayed a significantly higher peak of genomic PRRSV load in blood with a relative amount of 15.83 ± 2.63 (Log2 R) at 21 dpi (*p* = 0.0022) (Figure 2c). All contact piglets (6/6) became PRRSV positives in blood at 21 dpi demonstrating an efficient transmission of the vaccine strain from vaccinated to contact piglets. Regarding PRRSV genome quantification in nasal swabs, a significantly higher and longer PRRSV nasal shedding was recorded in contact pigs than in vaccinated animals (Figure S1) with a PRRSV load of 8.27 Log2 R for contact pigs at 21 dpi and 4.91 Log2 R at 4 dpi for vaccinated pigs and a mean shedding period of 22 and 12 days for contact and vaccinated pigs, respectively.

In this first trial, we evidenced in unvaccinated contact pigs an increased number of hyperthermia, as well as a significant decrease of growth performances. This clinical signs associated with a significant increase of the PRRSV viremia and nasal shedding suggested a partial loss of attenuation of the DV vaccine strain after transmission.

#### 3.1.2. Trial#2

**Figure 3:**
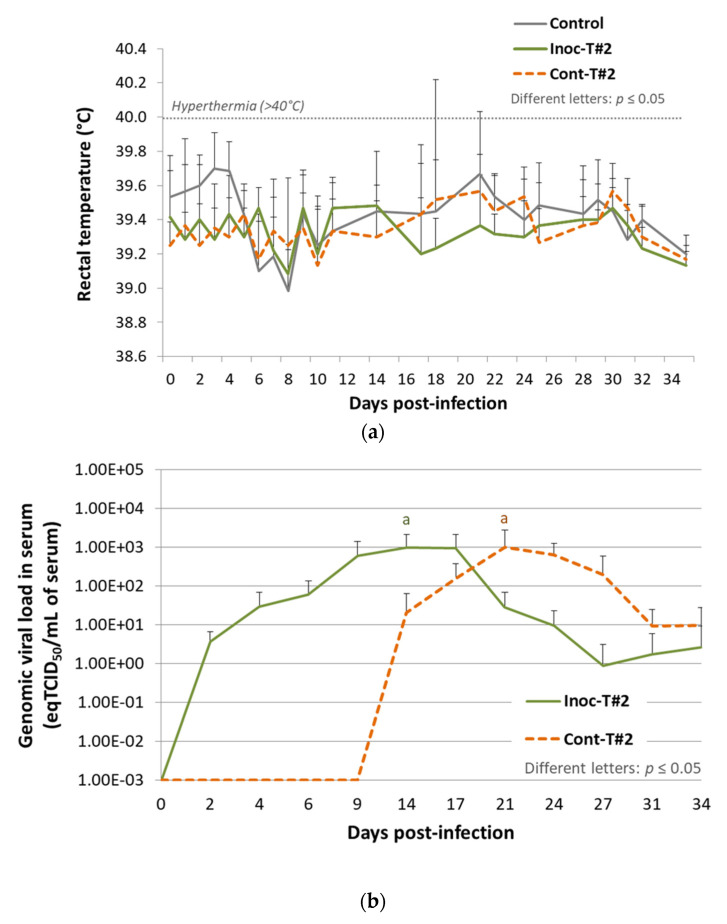
Results from Trial#2. (**a**) Evolution of the mean rectal temperatures (°C) in Inoc-T#2 inoculated pigs and in Cont-T#2 contact pigs after inoculation (day 0). Different letters indicate that the groups are significantly different from each other with *p* ≤ 0.05. (**b**) Evolution of the mean genomic viral loads in serum (eqTCID_50_/mL of serum) in Inoc-T#2 inoculated pigs and in Cont-T#2 contact pigs after inoculation (day 0). Different letters indicate that the groups are significantly different at peak values from each other with *p* ≤ 0.05.

In this second trial, we wanted to confirm the results of Trial#1 by using another batch of vaccine. Vaccinated (inoculated) and unvaccinated contact pigs from the 2017 trial did not show any significant increase of the mean rectal temperature compared to the control group throughout the study (Figure 3a).

Comparison of the average peak PRRSV genomic load in serum did not show any significant difference (*p* = 0.82) between vaccinated (10^2.99^ ± 10^3.06^ eqTCID_50_/mL at 14 dpi) and contact animals (10^3.00^ ± 10^3.25^ eqTCID_50_/mL at 21 dpi) (Figure 3b). However, all contact pigs (6/6) were detected PRRSV viremic at 21 dpi during the study, demonstrating the transmission of the vaccine strain from vaccinated to contact pigs.

To sum up, during Trial#2, we were able to confirm the transmission of the vaccine strain to all contact pigs as during Trial#1, but with no decrease of attenuation in the contact animals.

#### 3.1.3. Trial#3

In Trial#3, we aimed to confirm the increase of virulence of the DV strain shown in the contact animals of Trial#1. The virulence of a DV strain isolated from a diseased contact pig from Trial#1 (revertant strain: Rev) was thus compared to the virulence of a DV strain isolated from a healthy vaccinated pig (non-revertant strain: N-Rev) from the same trial.

##### Rectal Temperatures

**Figure 4:**
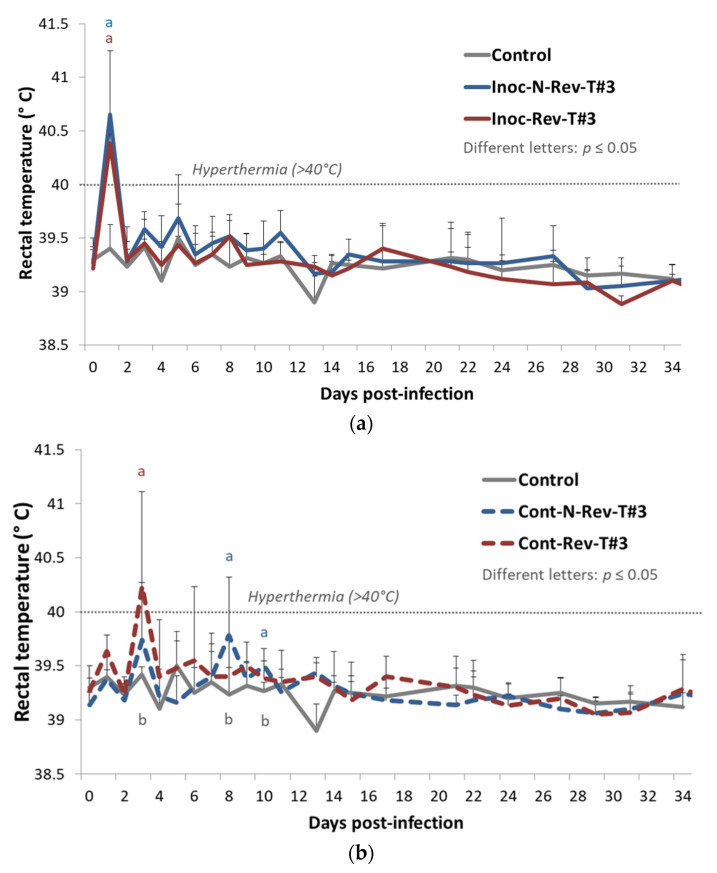
Evolution of the mean rectal temperatures (°C) in inoculated pigs (**a**) and contact pigs (**b**) after inoculation (day 0) during Trial#3. Different letters (“a” and “b”) indicate that the groups are significantly different from each other with *p* ≤ 0.05.

Inoc-N-Rev-T#3 (*p* = 0.016) and Inoc-Rev-T#3 (*p* = 0.015) inoculated pigs showed a significant hyperthermia (>40 °C) at 1 dpi compared to control animals, and then rectal temperatures from both inoculated groups became normal, i.e., they had the same value as for the control group (Figure 4a). For contact pigs, contact (Cont)-Rev-T#3 animals exhibited a significant hyperthermia (*p* = 0.048) at 3 dpi (2 days post-contact) compared to control animals whereas Cont-N-Rev-T#3 pigs demonstrated significant increases of the rectal temperature under the hyperthermia threshold (<40 °C) at 8 and 10 dpi (7 and 9 days of contact) (*p* = 0.049 and *p* = 0.022, respectively) compared to the control group (Figure 4b).

##### Viremia in Inoculated Pigs

**Figure 5:**
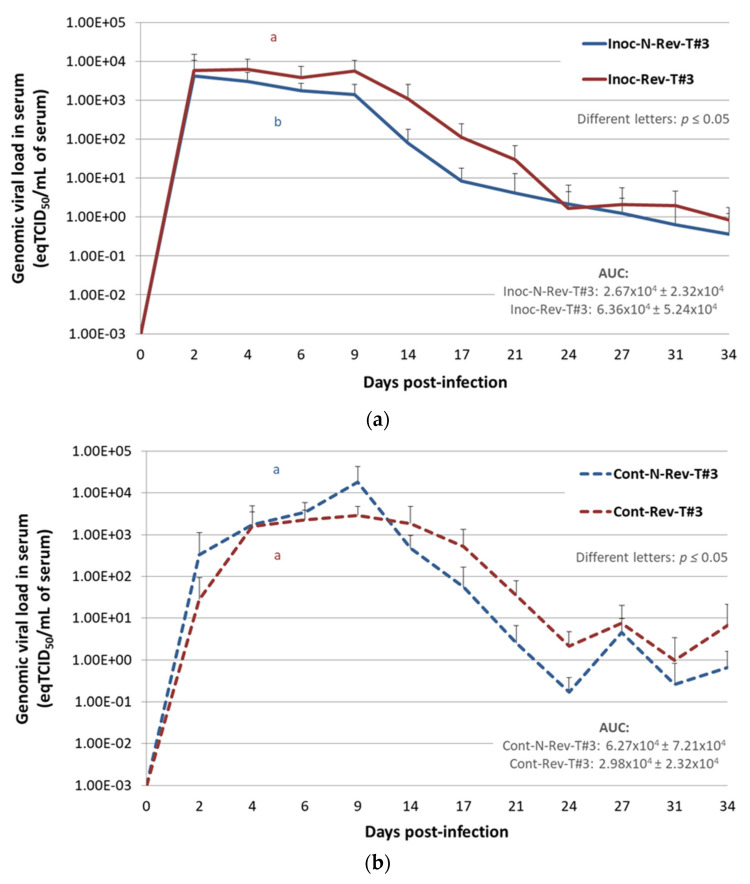

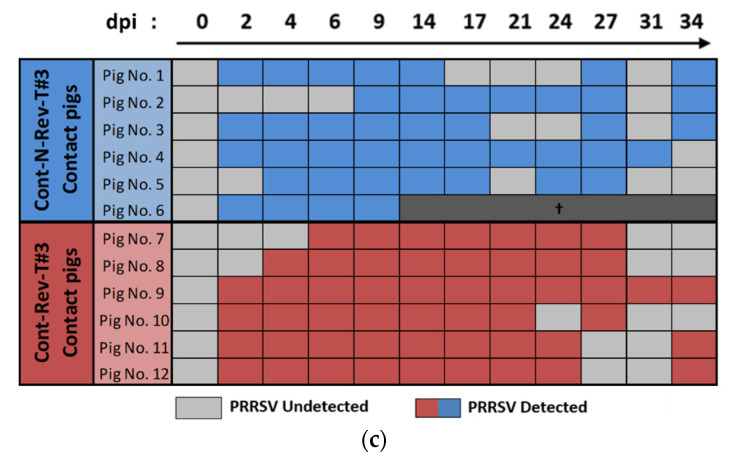
Porcine reproductive and respiratory syndrome virus (PRRSV) genomic quantification or detection in serum of inoculated and contact pigs during Trial#3. Evolution of the mean genomic viral loads in serum (eqTCID50/mL of serum) in inoculated non-revertant Trial#3 (Inoc-N-Rev-T#3) and inoculated revertant Trial#3 Inoc-Rev-T#3 inoculated pigs (**a**) and in Cont-N-Rev-T#3 and Cont-Rev-T#3 contact pigs (**b**) after inoculation (day 0) during Trial#3. Different letters (“a” and “b”) indicate that the groups are significantly different from each other after comparison of area under the curve (AUC) values with *p* ≤ 0.05. Data obtained from the Cont-N-Rev-T#3 pig euthanized at 10 dpi were not included in the average viral load AUC in serum. (**c**) Individual detections of viral genomes in the serum of Cont-N-Rev-T#3 and Cont-Rev-T#3 contact pigs.

For Inoc-Rev-T#3 pigs a viremia plateau was early reached between 2 and 9 dpi with a maximal mean viremia at 4 dpi showing 10^3.80^ ± 10^3.73^ eqTCID_50_/mL of serum, then the genomic viral load started to decrease from 9 to 34 dpi (Figure 5a). Inoc-N-Rev-T#3 animals reached a slightly lower mean viremia peak with 10^3.63^ ± 10^3.81^ eqTCID_50_/mL of serum at 2 dpi and the genomic PRRSV load decreased then gradually from 2 to 34 dpi. However, at the end of the study at 34 dpi, PRRSV genome was still detected in inoculated animals for both groups. Throughout the study, from 2 to 34 dpi, genomic viral loads in serum from Inoc-Rev-T#3 pigs were always higher than viremia of Inoc-N-Rev-T#3 pigs, with a significantly higher average viremia AUC for Inoc-Rev-T#3 pigs (*p* = 0.013).

##### Viral Nasal Excretion in Inoculated Pigs

PRRSV nasal excretion was detected from 2 to 14 dpi in Inoc-Rev-T#3 pigs and from 2 to 17 dpi in Inoc-N-Rev-T#3 pigs (Figure S2). Peak mean viral load in nasal swab supernatants were recorded both at 6 dpi with equivalent relative amounts of 4.13 ± 5.83 (Log2 R) and 4.97 ± 4.18 (Log2 R) for Inoc-Rev-T#3 and Inoc-N-Rev-T#3 pigs, respectively. Nasal viral shedding profiles were similar between both groups displaying no significantly different mean AUC values (*p* = 0.82).

##### Viremia in Contact Pigs

Excluding the Cont-N-Rev-T#3 pig euthanized at 10 dpi (pig No. 6), comparison of the average viremia AUC for Cont-N-Rev-T#3 and Cont-Rev-T#3 pigs did not reveal significant differences (*p* = 0.065) (Figure 5b). Regarding individual PRRSV genomic detections in serum, contact pigs started to become viremic from 2 dpi (1 day after contact with inoculated pigs) and all of them (6/6 per group) were PRRSV infected at 6 (5 days of contact) and 9 (8 days of contact) dpi in Cont-Rev-T#3 and Cont-N-Rev-T#3 groups, respectively (Figure 5c).

##### Transmission Parameters

Transmission parameters including daily transmission rate and duration of latency were estimated during Trial#3 for N-Rev and Rev strains. Both N-Rev and Rev strains displayed the same duration of latency: 0.7 days [0.4; 1.5] (Table 2). However, the transmission rate for the Rev strain (0.75 [0.29; 1.90]) was significantly higher than for the N-Rev strain (0.58 [0.23; 1.69]).

### 3.2. Genomic Characterization

#### 3.2.1. Coexistence of Different PRRSV Forms in Both DV Vaccine Batches

**Figure 6:**
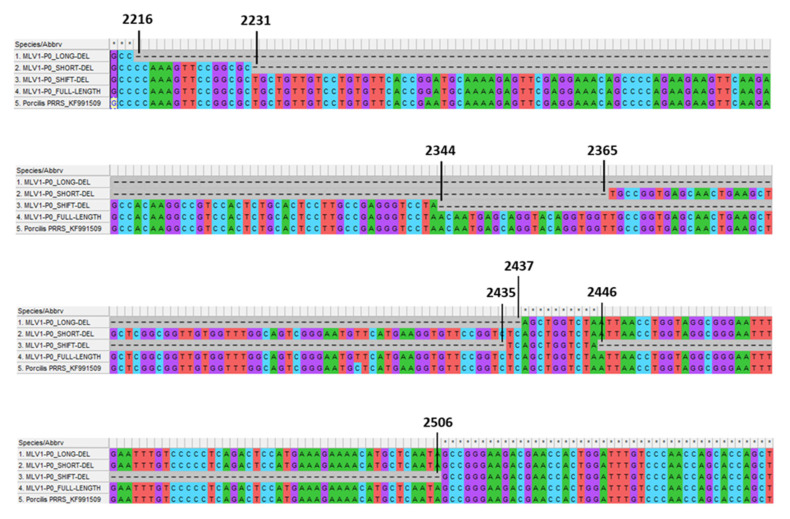
Alignment of the four PRRSV forms (three deletion variants and one full-length sequence) found in both Porcilis^®^ PRRS batch No. A142DB01 (Trial#1) and batch No. A207CB01 (Trial#2) with the KF991509.2 full-genome sequence of Porcilis^®^ PRRS DV strain as reference. Alignment targeted nucleotide positions from 2213 to 2545, including the deleted region (position No. starting from the first nt of 5′ nontranslated region (5′NTR)).

Prior to any experimentation a sample from Porcilis^®^ PRRS vaccine was amplified on MARC-145 cells to increase the amount of PRRSV nucleic acids before sequencing to improve the deepness of sequencing and robustness of the results. The mean coverage of sequencing per nucleotide for this sample was 482. The de novo assemblies with SPAdes and MIRA algorithms produced two different results. Compared to the initial reference (KF991509.2) both of the assembler results had a deletion which was 222 nucleotides (nt) long for the SPAdes contig (P0-LONG-DEL from nt 2216 to 2437) and 135 nt long for the MIRA assembly (P0-SHORT-DEL from nt 2231 to nt 2365) (Table 3 and Figure 6). Retrospectively, a minor third deletion, not identified in the de novo assemblies was also observed. This deletion (P0-SHIFT-DEL), which was 153 nt long, spanned from nt 2344 to 2435 then from nt 2446 to 2506 (Table 3 and Figure 6). An alignment on reference KF991509.2 was performed with bwa and the result of this alignment was used for an estimation of the distribution of the different variants of PRRSV genomes present in the sample by counting the reads corresponding to the different forms described around the deleted zone (i.e., nt 2216 to 2506). Interestingly, even if not observed in SPAdes or MIRA assembly, few reads corresponding to nt 2216–2437 (the deleted portion of the P0-LONG-DEL genome) were present leading to a fourth different form of PRRSV genome in the sample corresponding to the full-length genome (referring to KF991509.2).

To exclude any artifact resulting from the MARC-145 cell amplification, the PRRSV was directly sequenced from the vaccine vials used in Trial#1 and Trial#2. The deepness for the sequencing from the vaccine vial was much lower as compared to MARC-145 amplification, with a mean nt coverage of 87 for Trial#1 and 124 for Trial#2 vaccines. The FULL-LENGTH, LONG-DEL, SHORT-DEL and SHIFT-DEL forms were detected in the vaccine batch from Trial#2. For the vaccine batch used in Trial#1, the SHIFT-DEL variant was not detected, most probably due to the lower sequence coverage.

Even if the variation observed in the distribution of the different forms might only reflect the difference in coverage and not a strict variation in distribution, we tried to estimate the proportion of the different variants in the vaccines batch. We could thus consider that both vaccine batches were composed approximatively of more than 50% of the LONG-DEL variant, 30% of the SHORT-DEL variant and less than 10% of the SHIFT-DEL variant and of the FULL-LENGTH form.

#### 3.2.2. Selection of PRRSV Forms from Both DV Vaccine Batches Following Passages in Pigs

During Trial#1, the vaccinated animals were inoculated with a vaccine batch containing a mix of the four forms described previously (three deleted variants and one full-length form). In the vaccinated pigs, if considering the forms with reads count ≥ 1%, only one variant was detected in 4/6 pigs (2/4 pigs with LONG-DEL variant, 1/4 pig with SHORT-DEL variant and 1/4 pig with SHIFT-DEL variant) (Table 4). In the two remaining pigs, the coexistence of two variants was detected (two pigs co-infected with the SHORT-DEL and SHIFT-DEL variants). In all the contact pigs of Trial#1, only one form was detected in each pigs (4/5 pigs with LONG-DEL variant and 1/5 pig with SHORT-DEL variant). The SHIFT-DEL variant was not identified in any contact pig from Trial#1.

As in Trial#1, vaccinated pigs from Trial#2 also received a mix of the four PRRSV forms. Considering here also the forms with reads count ≥1%, the majority of inoculated pigs (5/6) owned one variant (2/5 pigs with LONG-DEL variant and 3/5 pigs with SHORT-DEL variant) (Table 4). Only 1/6 vaccinated pig displayed the presence of three forms (FULL-LENGTH form and LONG-DEL and SHORT-DEL variants). Focusing on contact pigs of Trial#2, as observed in Trial#1, all animals (6/6) owned only one variant (4/6 pigs with LONG-DEL variant and 2/6 pigs with SHORT-DEL variant). The SHIFT-DEL variant was not identified in both inoculated and contact pigs from Trial#2.

In Trial#1 and Trial#2, despite the presence of the four PRRSV forms in both vaccine batches, the number of forms following vaccination and transmission decreased with the number of passages in pigs. Indeed, only 3/11 inoculated pigs and 0/12 contact pigs displayed the coexistence of two forms (Table 4). Furthermore, in both trials, the FULL-LENGTH sequence represented a very minor form, compared to the deleted variants, and was found in only 2/23 animals (one contact pig from Trial#1 with very few reads and one inoculated pig from Trial#2). Globally, the two variants that are found in the majority of the animals (LONG-DEL and SHORT-DEL) are those that were estimated with the higher proportion in each vaccine batch.

#### 3.2.3. Identification of Non-Conservative Mutations during In Vivo Passages of DV Vaccine Strain

**Figure 7:**
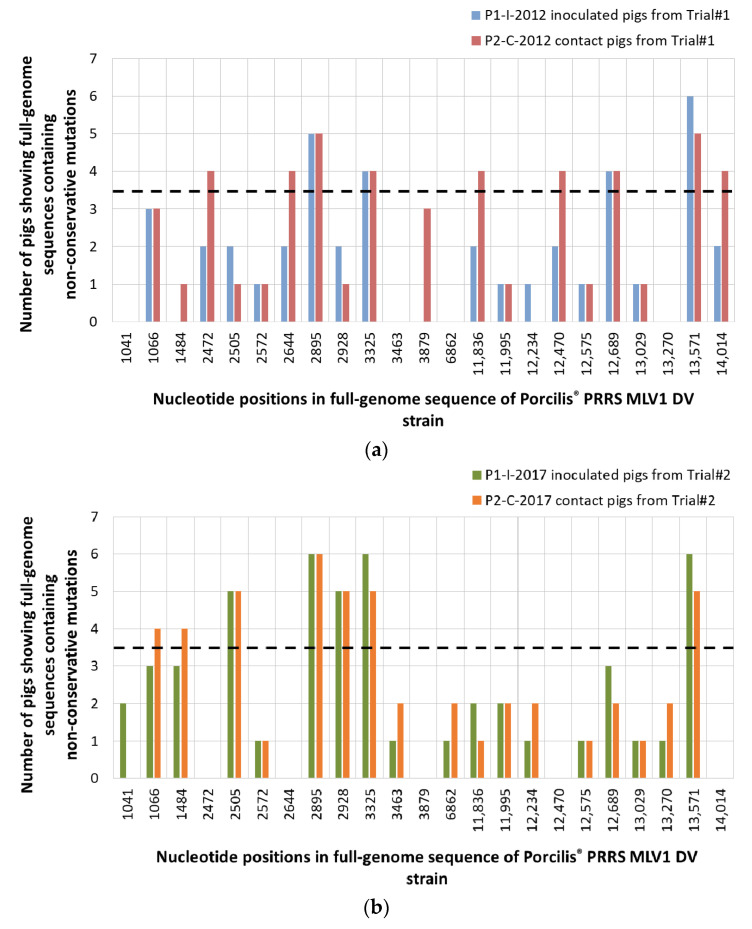
Distribution of the non-conservative mutations among inoculated and contact pigs from Trial#1 (**a**) and from Trial#2 (**b**), using the P0-2012 or the P0-2017 sequences as references, respectively. Mutation locations were indicated in the nucleotide sequence starting from the first nt of the 5′ nontranslated region (5′NTR) on the KF991509.2 full-length sequence. Mutations were considered as significant when occurred in at least 4/6 pigs (dotted line).

**Figure 8:**
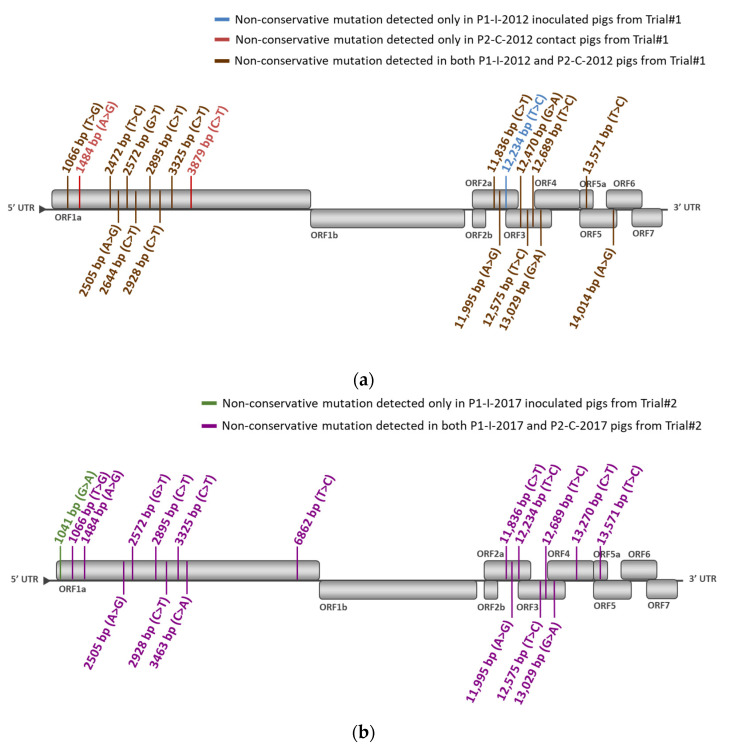
Localization in the PRRSV-1 genome of the identified non-conservative mutations occurred in P1-I-2012 and P2-C-2012 pigs during Trial#1 (**a**) or in P1-C-2017 and P2-C-2017 pigs during Trial#2 (**b**), using, respectively, the P0-2012 or the P0-2017 sequences as references. Mutation locations were indicated in the nucleotide sequence starting from the first nt of the 5′ nontranslated region (5′NTR) on the KF991509.2 full-length sequence and the start codon of each open reading frame (ORF), respectively.

Comparing between P0-2012 sequence (from the vaccine batch No. A142DB01) and P1-I-2012 sequences from vaccinated pigs from Trial#1, we found 12 non-conservative mutations in at least 2/6 inoculated pigs, including four mutations present in at least 4/6 pigs (Figure 7a, Figure 8a and Figure S3). The mutation located in ORF5a in nt position 13,571 was present in all (6/6) vaccinated pigs from Trial#1.

**Figure 9:**
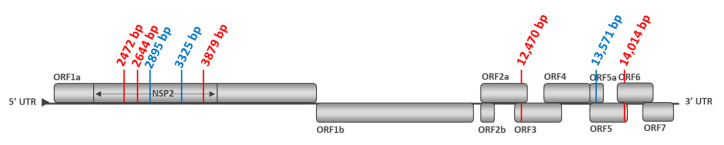
Localization in the PRRSV-1 genome of the identified mutations potentially linked to the adaptation (colored in blue) or the loss of attenuation (colored in red) phenomenon of the Porcilis^®^ PRRS DV strain after limited passages in pigs during Trial#1 and Trial#2. The KF991509.2 full-genome sequence of Porcilis^®^ PRRS modified live vaccine of type 1 (MLV1) DV strain is used as reference.

For Trial#2, comparison between P0-2017 sequence (from the vaccine batch No. A207CB01) and P1-I-2017 sequences from vaccinated pigs showed 11 non-conservative mutations detected in at least two pigs, including five mutations present in at least 4/6 pigs (Figure 7b, Figure 8b and Figure S4). Among the mutations found in the vaccinated pigs in both trials, three of them were present in almost all pigs: in nt position 2895 bp and 3325 bp in ORF1a and 13,571 bp in ORF5a. These mutations shared between both experiments could be potentially linked to the re-adaptation of the vaccine strain to the pig host following vaccination (Figure 9).

We then evaluated the PRRSV sequence evolution after natural transmission of the DV strain by comparing sequences from vaccinated and contact pigs from Trial#1. P1-I-2012 and P2-C-2012 sequences displayed the same 16 mutations with the exception of an additional non-conservative mutation located in ORF1a at 3879 bp present in three contact pigs (Figure 7a and Figure 8a). This mutation could be linked to further adaptation of the vaccine strain in pigs, or to the loss of attenuation of the DV strain seen in the contact pigs from Trial#1 (Figure 9). 

For Trial#2, comparison between P0-2017 and P2-C-2017 sequences from contact pigs led to the identification of the same mutations identified in P1-I-2017 sequences from vaccinated pigs, except for one mutation localized in ORF1a at 1041 bp which was absent in all contact pigs (Figure 7b and Figure 8b). No additional mutation appeared during transmission of the DV strain from vaccinated to contact pigs during Trial#2. 

Finally, as P2-C-2012 sequences were associated with a partially revertant phenotype, we compared them to P2-C-2017 sequences, which were not associated with reversion. Thus, nine different non-conservative mutations were found between P2-C-2012 and P2-C-2017 sequences (Figure 7a,b, Figures S3 and S4). Among them, 5/9 mutations were mostly encountered in P2-C-2012 sequences and located in ORF1a at 2472 bp, at 2644 bp and at 3879 bp; in ORF3 at 12,470 bp; and in ORF5 at 14,014 bp (Figure 8a,b). These five non-conservative mutations present only in P2-C-2012 sequences could be linked to the partial loss of attenuation of the DV strain observed in contact pigs from Trial#1 (Figure 9).

## 4. Discussion

In this study, we assessed the phenotype evolution of the DV vaccine strain after two consecutive passages in pigs and identified potential mutations linked to the adaptation to pig and to the virulence recovery of the vaccine DV strain. After deep sequencing of two batches of Porcilis^®^ PRRS MLV1, we were able to characterize four coexisting PRRSV genomic forms, namely one full-length sequence and three deleted variants characterized by different deletion patterns in ORF1. To the best of our knowledge, this study provided the first report combining a phenotypic and genetic analysis of the (i) re-adaptation of an MLV1 strain to pigs and of the (ii) partial loss of attenuation of this MLV1.

Starting with the phenotypic analysis, despite a quasi-similar genetic identity between both Porcilis^®^ PRRS batches, results obtained from Trial#1 and Trial#2 were surprisingly different. Indeed, a partial loss of attenuation of the DV vaccine strain occurred after only two passages in Trial#1, with contact pigs displaying more hyperthermia, lower ADWG and increased PRRSV viremia and nasal shedding than vaccinated pigs. During Trial#3, we were able to reproduce partially the results obtained in Trial#1 with significantly higher viremia for Inoc-Rev-T#3 pigs than for Inoc-N-Rev-T#3 animals. In contrast to Trial#1, no difference was observed between vaccinated and unvaccinated contact pigs during Trial#2, suggesting that the partial loss of attenuation of the DV strain evidenced during Trial#1 could be a seldom event.

Comparing viremia profiles from Inoc-T#1 with Inoc-N-Rev-T#3 and Inoc-Rev-T#3 inoculated pigs brought out that increased passage number was linked to faster in vivo replication of the DV-like strain in pigs. Concerning PRRSV nasal excretion, no significant difference was demonstrated between both inoculated groups from Trial#3. However, nasal viral shedding of DV-like vaccine strain for both passage two (from Inoc-N-Rev-T#3 group) and passage three (from Inoc-Rev-T#3 group) was higher and lasted longer than in vaccinated pigs with passage one, as previously shown in an another study using the same experimental setup and quantification method [20]. This strong viral nasal excretion may support the important transmission rate observed in both groups from Trial#3. The more passages in pigs, the higher the transmission. Indeed, the daily transmission rate of the passage one of DV vaccine strain, previously determined by using the same model, was 0.11 [0.05; 0.22] infected pigs by one infectious pig per day [20]. In comparison, with P2 and P3, the daily transmission rate was five to seven times higher for the DV-like N-Rev and DV-like Rev strains, respectively. These daily transmission rates are higher to the one we previously evaluated for a PRRSV-1 field strain [21,23]. Khatun et al., in an evaluation of the phenotypic stability during three pig-to-pig passages of a MLV2 demonstrated in the second and the third passage that PRRSV genome loads in sera and tissues were dramatically increased as compared to the first passage [24]. In another recently published study [25], we compared clinical, virological and transmission parameters between a Unistrain^®^ PRRS vaccinated group and a Unistrain-like strain inoculated group. The Unistrain-like strain came from the field where few passages in pigs were completed, and even if it displayed few clinical signs, it resulted in significantly increased viremia and transmission compared to vaccinated pigs. Number of studies from the field reported safety issues of MLVs due to virulence recovery [26]. After the accumulation of several non-conservative mutations following multiple passages in pigs, MLV2 strains can revert to virulence state in the field, inducing clinical signs comparable to those induced by a wild-type PRRSV infection [14].

Moving to genetic analysis, we were able to identify the presence in both batches of Porcilis^®^ PRRS vaccine used during Trial#1 and Trial#2 of three to four PRRSV forms, including one full-length sequence and three variants presenting three different deletion profiles localized in ORF1a (NSP2). In a study from 2014, the DV vaccine strain was full-genome sequenced and deposited in GenBank, under the accession No. KJ127878. This PRRSV sequence presents a deletion between 2216 and 2437 nt identical to the long-deleted variant that we identified as the major form in both Porcilis^®^ PRRS batches [27]. The existence of PRRSV variants in Porcilis^®^ PRRS was previously described in 2015, in another study [28]. In this study, partial sequencing of Porcilis^®^ PRRS revealed the presence of a mix of two variants presenting deletions of 222 and 135 nt in NSP2, strongly similar to the long- and short-deleted variants that we identified. Following inoculation of these variants through vaccination and then through horizontal transmission, no variant seemed being preferentially selected in pigs since variant frequencies in inoculated and contact pigs from both trials were similar to estimated variant frequencies determined in both Porcilis^®^ PRRS vaccine batches sequenced. The variants transmission profile showed in our trials was in concordance with the main results from Cortey et al., who demonstrated a reduction in the variant diversity after a transmission event, (genetic bottleneck) during PRRSV-1 transmission [29]. Indeed, coexistence of three viral forms was detected in only one vaccinated pig and the presence of two variants in two inoculated animals. After a further viral transmission, only one variant was detected for all the contact pigs. In our limited experimental conditions, we hardly demonstrated the transmission of the full-length sequence and the shift-deleted variant that were poorly represented in the different batches. However, the analysis of field samples from vaccinated herds allowed us to identify at least the three deleted variants described in this study for Porcilis^®^ PRRS vaccine, suggesting that all forms might be transmissible (Personal Communication).

Comparing the sequences of the MLV1 from passage 0 to passage 1, we were able to identify three mutations potentially linked to the adaptation of the DV strain in vaccinated pigs. These mutations were present in >75% of pigs and were localized in ORF1a at 2895 bp and 3325 bp both in the encoding region for the NSP2 and in ORF5a at 13,571 bp encoding for the 5a minor structural protein. Some studies previously identified genetic determinants linked to the attenuation of PRRSV-2 strains following multiple cell passages. Similarly, to our results, the majority of these mutations were localized in ORF1a and especially in NSP1 and NSP2 [30,31,32]. The authors suggested that these mutations in NSP1 and NSP2 encoding regions could impact the efficacy level of the NSP proteolytic cleavage compromising the viral replication. In our case, the mutations in NSP2 due to the adaptation of the DV strain in pigs may be linked to the increased in vivo replication efficacy observed during passages in pigs. Indeed, Song J. et al. [33], revealed a link between the NSP2 region and the viral tropism. After testing mutant variants in this hypervariable region, deleted HP-PRRSV-2 strain was found to loose infectivity in PAMs and failed to establish an infection in piglets. In contrast to previous studies, we did not identify adaptive mutations in ORF5 and ORF7 coding for GP5 and N proteins, respectively [30,34,35,36]. Another study discovered a triple amino acid substitution in GP2a as a determining factor in PRRSV-1 adaptation to MARC-145 cells, demonstrating improved growth characteristics in cell-culture for strains presenting the triple substitution [37]. However, the authors compared MARC-145 adapted virus sequences with the sequences of 70 field PRRS viruses which have been adapted to pigs after several in vivo passages. Therefore, we can further speculate that the vaccine-like strains we characterized may not accumulate enough passages in pigs to gain mutations in the GP2 coding gene. In our study, most of the adaptive mutations appeared during the first passage in pigs, following vaccination. Similarly to us, Grebennikova et al., demonstrated that a wild-type PRRSV-2 strain previously attenuated after more than 200 passages in cell culture, accumulated six non-conservative mutations localized in ORF1a, ORF1b and ORF6 regions and a partial reversion to virulence after only one passage in pigs [14].

We also identified five mutations potentially related to the partial loss of attenuation of the DV vaccine strain encountered in most of contact pigs from Trial#1. Three mutations were located in ORF1a (coding region for NSP2), one was situated in ORF3 (coding region for GP3) and the last one in ORF5 (coding region for GP5). Individual data from Trial#1 showed that almost all the pigs infected with the strain showing the five mutations displayed hyperthermia and highest and longest viral loads in blood from their groups. Conversely, the only contact pig from Trial#1 that did not display these mutations demonstrate no hyperthermia and low PRRSV viremia. As expected, the P1-I-2012 strain (sequence from pig No. 2012_7) isolated from a vaccinated pig from Trial#1 and used as inoculum for the Inoc-N-Rev-T#3 inoculated pigs did not present any of these five mutations. In contrast, the P2-C-2012 strain sequence (from pig No. 2012_6) isolated from a diseased contact pig, and used as inoculum for the Inoc-Rev-T#3 inoculated pigs displayed all the five mutations potentially linked to the attenuation loss. In Denmark, following a large vaccination program using a MLV2, several naïve pig herds became infected with a MLV2-like strain reverted to virulence and associated with disastrous reproductive troubles [12,38,39,40]. Five mutations independently occurred in three MLV2-like strains in the field. Contrasting to our results, two mutations, directly linked to reversion, were localized in ORF1a in the NSP1β and in the NSP10. However, many authors could not exclude the existence of other mutations linked to the loss of attenuation of MLVs in other ORFs [12,14,30,34]. Multiple modifications found in NSP2 in various PRRSV-1 and PRRSV-2 strains have been linked to different phenotypes, including NSP2 mutations probably involved in the loss of attenuation and virulence recovery of MLV strains [41]. Finally, all of these studies aiming at the identification of PRRSV genetic determinants of attenuation, adaptation or attenuation loss tend to support the idea that genetic factors linked to the virulence level of MLV strains are probably multigenic and localized in encoding regions for both structural and non-structural proteins [15,42].

It should also be kept in mind that accumulation of mutations due to multiple passages in pigs may not be the only genetic factors contributing to the loss of attenuation and reversion to virulence of vaccine strains. Recently, a Danish team revealed the existence of a PRRSV-1 recombinant strain between two MLV1 strains from Unistrain^®^ PRRS and Suvaxyn^®^ PRRS MLV vaccines [43]. This recombinant vaccine PRRSV-1 strain caused more severe disease in infected herds when compared to the clinical impact of other field PRRSV-1 strains and despite the high level of genetic identity shared with attenuated parent vaccine strains. These findings suggested that recombination may also generate new strains with higher virulence.

To conclude, we were able to show in this study that the PRRSV MLV1 DV strain is easily transmitted from inoculated to contact pigs. After one additional passage in pig, these transmission capacities are further increased and reach or even surpass those of a PRRSV-1 field strain. We also showed that the transmission of the vaccine strain could more rarely be associated with a partial loss of attenuation. At the genetic point of view, the re-adaptation of the vaccine strain to the pig host is associated with some specific mutations that are very rapidly acquired.

In the field, the vaccination protocols should be optimized so that the transmission of PRRSV MLV1 strains to naïve pigs, their circulation and persistence in pig herds are minimized in order to prevent a possible virulence recovery. From this point of view, the vaccination of all the animals in the same batch/unit should be recommended to prevent PRRSV MLV1 transmission and reversion.

## Figures and Tables

**Table 1:** Experiment parameters for Trial#1, Trial#2 and Trial#3.

Trial	Trial#1	Trial#2	Trial#3	
**Number of pigs**	6 inoculated 6 contact	6 inoculated 6 contact	6 inoculated 6 contact	6 inoculated 6 contact
**Age of pigs** (weeks)	6	7	7	7
**Inoculated strain**	Porcilis^®^ PRRS (batch No. A142DB01) P0-2012	Porcilis^®^ PRRS (batch No. A207CB01) P0-2017	DV isolate P1-I-2012	DV isolate P2-C-2012
**Inoculum titer in MARC-145 cells** (TCID_50_/mL)	10^4.2^	10^4.8^	10^4.5^	10^4.5^
**Inoculation route**	IM	IM	IM	IM

PRRS, porcine reproductive and respiratory syndrome; TCID, tissue culture infectious dose; IM, intramuscularly.

**Table 2:** Estimation of transmission parameters from Inoc-N-Rev-T#3 inoculated pigs (passage two) to Cont-N-Rev-T#3 contact pigs (passage three) and from Inoc-Rev-T#3 inoculated pigs (passage three) to Cont-Rev-T#3 contact pigs (passage four).

Transmission Parameters	N-Rev Strain	Rev Strain
**Daily transmission rate** (number of infected pigs per one infectious pig per day)	0.58 ^a^ [0.23; 1.69] *	0.75 ^b^ [0.29; 1.90]
**Duration of latency** (days)	0.7 ^a^ [0.4; 1.7]	0.7 ^a^ [0.4; 1.5]

* 95% credibility interval. Different letters (“^a^” and “^b^”) indicate that the groups are significantly different from each other with *p* ≤ 0.05.

**Table 3:** Location and size of deletions in the full-genome sequences for the three different variants found in both Porcilis^®^ PRRS vaccine batches, and their percentage of identity compared to the sequence No. KF991509.2 of Porcilis^®^ PRRS DV strain.

Variant Name	Deletion Position (Nucleotide Base Pair)	Deletion Size (Base Pair)	Identity Rate with KF991509.2 (%)
P0-LONG-DEL	2216–2437	222	99.95
P0-SHORT-DEL	2231–2365	135	99.93
P0-SHIFT-DEL	2344–2435 2446–2506	92 61	99.94

**Table 4:** Selection of PRRSV forms (full-length sequence and variants) from Porcilis^®^ PRRS vaccine after vaccinations and transmissions during Trial#1 and Trial#2.

Trial	Inoculated or Contact Pig	Pen	Pig No.	Full-Length Sequence	Variants			% Identity with Variants of Reference (P0)
					Long(L)	Short(S)	Shift(F)	
**#1**	Inoc	1	2012_1	ND	<1%	>99%	ND	99.79%
	Inoc	1	2012_2	ND	>99%	ND	ND	99.87%
	Inoc	1	2012_3	ND	ND	32%	67%	99.86%
	Cont	1	2012_4	<1%	>99%	ND	ND	99.85%
	Cont	1	2012_5	ND	>99%	ND	ND	99.85%
	Cont	1	2012_6 **	ND	>99%	ND	ND	99.84%
	Inoc	2	2012_7 *	ND	ND	ND	>99%	99.87%
	Inoc	2	2012_8	ND	>99%	ND	ND	99.85%
	Inoc	2	2012_9	ND	ND	50%	50%	99.90%
	Cont	2	2012_10	ND	>99%	ND	ND	99.86%
	Cont	2	2012_11	ND	ND	>99%	ND	99.85%
Viral form detection frequency in Inoc pigs (≥1%)				0/6	2/6	3/6	3/6	
Viral form detection frequency in Cont pigs (≥1%)				0/5	4/5	1/5	0/5	
Viral form detection frequency in Trial#1 (≥1%)				0/11	6/11	4/11	3/11	
**#2**	Inoc	1	2017_12	ND	>99%	ND	ND	99.83%
	Inoc	1	2017_13	ND	ND	>99%	ND	99.83%
	Inoc	1	2017_14	ND	<1%	>99%	ND	99.85%
	Cont	1	2017_15	ND	<1%	>99%	ND	99.79%
	Cont	1	2017_16	ND	>99%	<1%	ND	99.82%
	Cont	1	2017_17	ND	<1%	>99%	ND	99.83%
	Inoc	2	2017_18	ND	ND	>99%	ND	99.78%
	Inoc	2	2017_19	ND	>99%	ND	ND	99.85%
	Inoc	2	2017_20	5%	44%	51%	ND	99.87%
	Cont	2	2017_21	ND	>99%	ND	ND	99.83%
	Cont	2	2017_22	ND	>99%	ND	ND	99.83%
	Cont	2	2017_23	ND	>99%	ND	ND	99.87%
Viral form detection frequency in Inoc pigs (≥1%)				1/6	3/6	4/6	0/6	
Viral form detection frequency in Cont pigs (≥1%)				0/6	4/6	2/6	0/6	
Viral form detection frequency in Trial#2 (≥1%)				1/12	7/12	6/12	0/12	
TOTAL VIRAL FORM DETECTION FREQUENCY (≥1%)				1/23	13/23	10/23	3/23	

Inoc, inoculated; Cont, contact; ND abbreviation is used for reads not determined. Identity rates of the major variant present in pigs were determined after an alignment by comparison between sequences from pig samples from Trial#1 and Trial#2 with P0-LONG-DEL, -SHORT-DEL or -SHIFT-DEL parental variants. * Isolate from the pig No. 2012_7 was used as inoculum in Inoc-N-Rev-T#3 inoculated pigs from Trial#3. ** Isolate from the pig No. 2012_6 was used as inoculum in Inoc-Rev-T#3 inoculated pigs from Trial#3.

## Data Availability

Data from the study are available upon reasonable request to the corresponding author. All the full-genome sequences listed in this article are gathered into the BioProject No. PRJNA705101.

## Supplementary Materials

The following are available online at https://www.mdpi.com/article/10.3390/vaccines9040392/s1. Table S1: Presentation of the vaccine strains isolated from vaccinated and contact pigs from Trial#1 and Trial#2. Figure S1: Evolution of the mean genomic viral loads (relative amount expressed in Log2 R) in inoculated Inoc-T#1 and contact Cont-T#1 pigs in nasal swab supernatants after inoculation (day 0) during Trial#1. Different letters (“a” and “b”) indicate that the groups are significantly different from each other after comparison of area under the curve (AUC) values with *p* ≤ 0.05. Figure S2: Evolution of the mean genomic viral loads (relative amount expressed in Log2 R) in Inoc-N-Rev-T#3 and Inoc-Rev-T#3 inoculated pigs in nasal swab supernatants after inoculation (day 0) during Trial#3. Different letters indicate that the groups are significantly different from each other after comparison of of area under the curve (AUC) values with *p* ≤ 0.05. Figure S3: Identification of non-conservative mutations in P1-I-2012 and P2-C-2012 sequences from inoculated and contact pigs from Trial#1 using the P0-2012 sequence as reference. Mutation location were indicated in nucleotide (nt) and amino acid (aa) sequences position, starting from the first nt of the 5’ nontranslated region (5’NTR) on the KF991509.2 full-length sequence and the start codon of each open reading frame (ORF), respectively. * Isolate from the pig No. 2012_7 was used as inoculum in Inoc-N-Rev-T#3 inoculated pigs from Trial#3. ** Isolate from the pig No. 2012_6 was used as inoculum in Inoc-Rev-T#3 inoculated pigs from Trial#3. Figure S4: Identification of non-conservative mutations in P1-I-2017 and P2-C-2017 sequences from inoculated and contact pigs from Trial#2 using the P0-2017 sequence as reference. Mutation location were indicated in nucleotide (nt) and amino acid (aa) sequences position, starting from the first nt of the 5’ nontranslated region (5’NTR) on the KF991509.2 full-length sequence and the start codon of each open reading frame (ORF), respectively.
