# Supplementary material for: Phenotypic and Genetic Evolutions of a Porcine Reproductive and Respiratory Syndrome Modified Live Vaccine after Limited Passages in Pigs
Source: Vaccines (Basel). 2021 Apr 16;9(4):392. doi: 10.3390/vaccines9040392 (PMC8073166; doi:10.3390/vaccines9040392)
Supplement: Supplementary file 1 [file vaccines-09-00392-s001.zip › vaccines-1144520-supplementary.pdf]

vaccines MDPI

Supplementary Materials:

Table **S1:** Presentation of the vaccine strains isolated from vaccinated and contact pigs from Trial#1 and Trial#2.

Trial	Inoculated or Contact pig	Pen	Pig No.	Dpi selected for DV strain isolation
#1	Inoc	1	2012_1	14
	Inoc	1	2012_2	10
	Inoc	1	2012_3	7
	Cont	1	2012_4	14
	Cont	1	2012_5	21
	Cont	1	2012_6 **	21
	Inoc	2	2012_7 *	14
	Inoc	2	2012_8	10
	Inoc	2	2012_9	10
	Cont	2	2012_10	17
	Cont	2	2012_11	24
#2	Inoc	1	2017_12	10
	Inoc	1	2017_13	14
	Inoc	1	2017_14	14
	Cont	1	2017_15	21
	Cont	1	2017_16	17
	Cont	1	2017_17	24
	Inoc	2	2017_18	14
	Inoc	2	2017_19	17
	Inoc	2	2017_20	17

	Cont	2	2017_21	24
	Cont	2	2017_22	21
	Cont	2	2017_23	24

The days post-infection (dpi) chosen for the DV strain isolation was selected at viremia peak for each pig. Dpi, days post-infection; inoc, inoculated; cont, contact. * Isolate from the pig No. 2012_7 was used as inoculum in Inoc-N-Rev-T#3 inoculated pigs from Trial#3. ** Isolate from the pig No. 2012_6 was used as inoculum in Inoc-Rev-T#3 inoculated pigs from Trial#3.

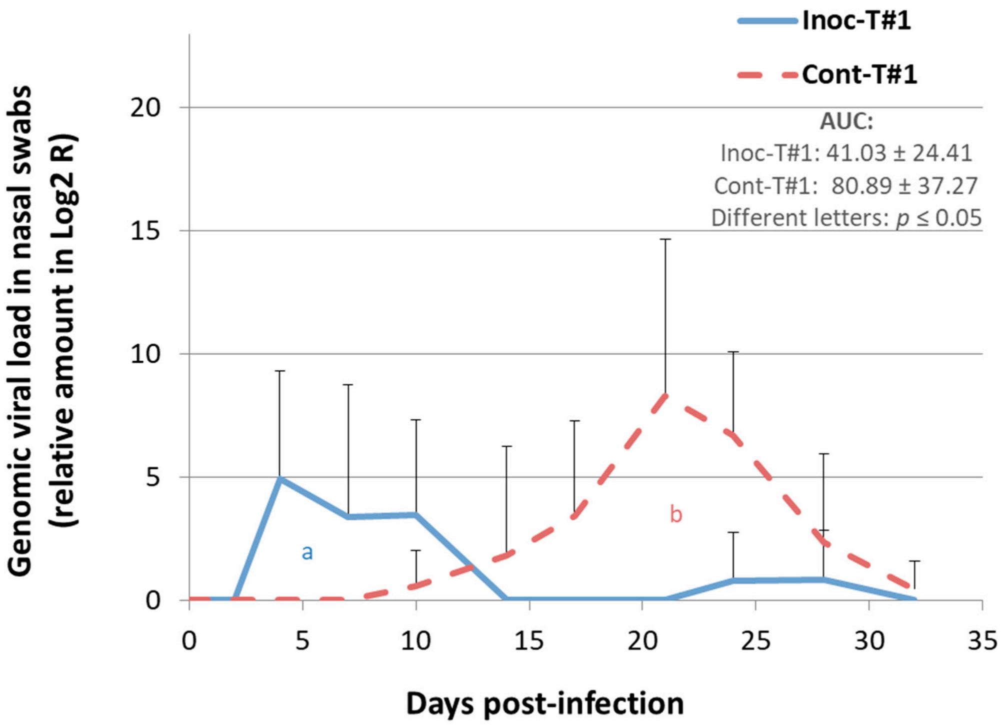

Figure S1: Evolution of the mean genomic viral loads (relative amount expressed in Log2 R) in inoculated Inoc-T#1 and contact Cont-T#1 pigs in nasal swab supernatants after inoculation (day 0) during Trial#1. Different letters ("a" and "b") indicate that the groups are significantly different from each other after comparison of Area Under the Curve (AUC) values with $p \leq 0.05$.

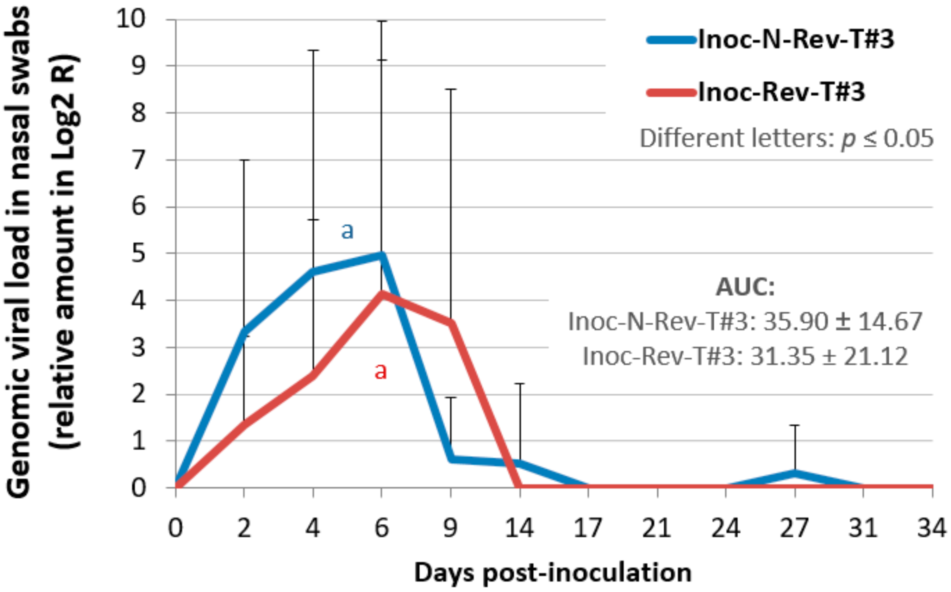

Figure S2: Evolution of the mean genomic viral loads (relative amount expressed in Log2 R) in Inoc-N-Rev-T#3 and Inoc-Rev-T#3 inoculated pigs in nasal swab supernatants after inoculation (day 0) during Trial#3. Different letters indicate that the groups are significantly different from each other after comparison of of Area Under the Curve (AUC) values with $p \leq 0.05$.

Gene	P1-I-2012												P2-C-2012									
	2012_1		2012_2		2012_3		2012_7 *		2012_8		2012_9		2012_4		2012_5		2012_6 **		2012_10		2012_11	
	nt	aa	nt	aa	nt	aa	nt	aa	nt	aa	nt	aa	nt	aa	nt	aa	nt	aa	nt	aa	nt	aa
ORF1a					T>G 1066	L>W 282			T>G 1066	L>W 282	T>G 1066	L>W 282	T>G 1066	L>W 282					T>G 1066	L>W 282	T>G 1066	L>W 282
ORF1a																					A>G 1484	T>A 425
ORF1a			T>C 2472	S>P 751					T>C 2472	S>P 751			T>C 2472	S>P 751	T>C 2472	S>P 751	T>C 2472	S>P 751	T>C 2472	S>P 751		
ORF1a	A>G 2505	N>D 762			A>G 2505	N>D 762															A>G 2505	N>D 762
ORF1a					G>T 2572	R>I 784															G>T 2572	R>I 784
ORF1a			C>T 2644	T>M 808					C>T 2644	T>M 808			C>T 2644	T>M 808	C>T 2644	T>M 808	C>T 2644	T>M 808	C>T 2644	T>M 808		
ORF1a	C>T 2895	P>S 892	C>T 2895	P>S 892	C>T 2895	P>S 892	C>T 2895	P>S 892	C>T 2895	P>S 892			C>T 2895	P>S 892	C>T 2895	P>S 892	C>T 2895	P>S 892	C>T 2895	P>S 892	C>T 2895	P>S 892
ORF1a	C>T 2928	P>S 903									C>T 2928	P>S 903									C>T 2928	P>S 903
ORF1a	C>T 3325	P>L 1035	C>T 3325	P>L 1035			C>T 3325	P>L 1035	C>T 3325	P>L 1035			C>T 3325	P>L 1035	C>T 3325	P>L 1035	C>T 3325	P>L 1035	C>T 3325	P>L 1035		
ORF1a													C>T 3879	R>C 1220	C>T 3879	R>C 1220	C>T 3879	R>C 1220				
1b																						
2a			C>T 11836	S>L 14					C>T 11836	S>L 14			C>T 11836	S>L 14	C>T 11836	S>L 14	C>T 11836	S>L 14	C>T 11836	S>L 14		
2a					A>G 11995	N>S 67															A>G 11995	N>S 67
2a	T>C 12234	Y>H 147																				
2b																						
3			G>A 12470	A>T 23					G>A 12470	A>T 23			G>A 12470	A>T 23	G>A 12470	A>T 23	G>A 12470	A>T 23	G>A 12470	A>T 23		
3											T>C 12575	S>P 58									T>C 12575	S>P 58
3	T>C 12689	S>P 96	T>C 12689	S>P 96	T>C 12689	S>P 96			T>C 12689	S>P 96			T>C 12689	S>P 96	T>C 12689	S>P 96	T>C 12689	S>P 96	T>C 12689	S>P 96		
3							G>A 13029	R>Q 266													G>A 13029	R>Q 266
4																						
5	T>C 13571	F>L 25	T>C 13571	F>L 25	T>C 13571	F>L 25	T>C 13571	F>L 25	T>C 13571	F>L 25	T>C 13571	F>L 25	T>C 13571	F>L 25	T>C 13571	F>L 25	T>C 13571	F>L 25	T>C 13571	F>L 25	T>C 13571	F>L 25
5a			A>G 14014	N>S 174					A>G 14014	N>S 174			A>G 14014	N>S 174	A>G 14014	N>S 174	A>G 14014	N>S 174	A>G 14014	N>S 174		
6																						
7																						

Figure S3: Identification of non-conservative mutations in P1-I-2012 and P2-C-2012 sequences from inoculated and contact pigs from Trial#1 using the P0-2012 sequence as reference. Mutation location were indicated in nucleotide (nt) and amino acid (aa) sequences position, starting from the first nt of the 5′ nontranslated region (5′NTR) on the KF991509.2 full-length sequence and the start codon of each open reading frame (ORF), respectively. * Isolate from the pig No. 2012_7 was used as inoculum in Inoc-N-Rev-T#3 inoculated pigs from Trial#3. ** Isolate from the pig No. 2012_6 was used as inoculum in Inoc-Rev-T#3 inoculated pigs from Trial#3.

P1-I-2017												P2-C-2017												ORF
2017_12		2017_13		2017_14		2017_18		2017_19		2017_20		2017_15		2017_16		2017_17		2017_21		2017_22		2017_23		
nt	aa	nt	aa	nt	aa	nt	aa	nt	aa	nt	aa	nt	aa	nt	aa	nt	aa	nt	aa	nt	aa	nt	aa	
								G>A<br>1041	G>S<br>274	G>A<br>1041	G>N<br>274													ORF1a
T>G<br>1066	L>W<br>282							T>G<br>1066	L>W<br>282	T>G<br>1066	L>W<br>282			T>G<br>1066	L>W<br>282			T>G<br>1066	L>W<br>282	T>G<br>1066	L>W<br>282	T>G<br>1066	L>W<br>282	ORF1a
A>G<br>1484	T>A<br>425	A>G<br>1484	T>A<br>425			A>G<br>1484	T>A<br>425					A>G<br>1484	T>A<br>425	A>G<br>1484	T>A<br>425					A>G<br>1484	T>A<br>425	A>G<br>1484	T>A<br>425	ORF1a
A>G<br>2505	N>D<br>762	A>G<br>2505	N>D<br>762	A>G<br>2505	N>D<br>762	A>G<br>2505	N>D<br>762			A>G<br>2505	N>D<br>762	A>G<br>2505	N>D<br>762	A>G<br>2505	N>D<br>762	A>G<br>2505	N>D<br>762	A>G<br>2505	N>D<br>762	A>G<br>2505	N>D<br>762			ORF1a
				G>T<br>2572	R>I<br>784											G>T<br>2572	R>I<br>784							ORF1a
C>T<br>2895	P>S<br>892	C>T<br>2895	P>S<br>892	C>T<br>2895	P>S<br>892	C>T<br>2895	P>S<br>892	C>T<br>2895	P>S<br>892	C>T<br>2895	P>S<br>892	C>T<br>2895	P>S<br>892	C>T<br>2895	P>S<br>892	C>T<br>2895	P>S<br>892	C>T<br>2895	P>S<br>892	C>T<br>2895	P>S<br>892	C>T<br>2895	P>S<br>892	ORF1a
C>T<br>2928	P>S<br>903	C>T<br>2928	P>S<br>903	C>T<br>2928	P>S<br>903	C>T<br>2928	P>S<br>903			C>T<br>2928	P>S<br>903	C>T<br>2928	P>S<br>903	C>T<br>2928	P>S<br>903	C>T<br>2928	P>S<br>903	C>T<br>2928	P>S<br>903	C>T<br>2928	P>S<br>903			ORF1a
C>T<br>3325	P>L<br>1035	C>T<br>3325	P>L<br>1035	C>T<br>3325	P>L<br>1035	C>T<br>3325	P>L<br>1035	C>T<br>3325	P>L<br>1035	C>T<br>3325	P>L<br>1035	C>T<br>3325	P>L<br>1035	C>T<br>3325	P>L<br>1035	C>T<br>3325	P>L<br>1035	C>T<br>3325	P>L<br>1035	C>T<br>3325	P>L<br>1035			ORF1a
C>A<br>3463	P>Q<br>1081													C>A<br>3463	P>Q<br>1081					C>A<br>3463	P>Q<br>1081			ORF1a
T>C<br>6862	L>P<br>2214													T>C<br>6862	L>P<br>2214					T>C<br>6862	L>P<br>2214			ORF1a
																								1b
		C>T<br>11836	S>L<br>14					C>T<br>11836	S>L<br>14			C>T<br>11836	S>L<br>14											2a
				A>G<br>11995	N>S<br>67	A>G<br>11995	N>S<br>67									A>G<br>11995	N>S<br>67	A>G<br>11995	N>S<br>67					2a
										T>A<br>12234	Y>N<br>147							T>C<br>12234	Y>H<br>147			T>C<br>12234	Y>H<br>147	2a
																								2b
				T>C<br>12575	S>P<br>58											T>C<br>12575	S>P<br>58							3
		T>C<br>12689	S>P<br>96					T>C<br>12689	S>P<br>96	T>C<br>12689	S>P<br>96	T>C<br>12689	S>P<br>96					T>C<br>12689	S>P<br>96					3
				G>A<br>13029	R>Q<br>266											G>A<br>13029	R>Q<br>266							3
C>T<br>13270	H>Y<br>109													C>T<br>13270	H>Y<br>109					C>T<br>13270	H>Y<br>109			4
																								5
T>C<br>13571	F>L<br>25	T>C<br>13571	F>L<br>25	T>C<br>13571	F>L<br>25	T>C<br>13571	F>L<br>25	T>C<br>13571	F>L<br>25	T>C<br>13571	F>L<br>25	T>C<br>13571	F>L<br>25	T>C<br>13571	F>L<br>25	T>C<br>13571	F>L<br>25	T>C<br>13571	F>L<br>25	T>C<br>13571	F>L<br>25			5a
																								6
																								7

Figure S4: Identification of non-conservative mutations in P1-I-2017 and P2-C-2017 sequences from inoculated and contact pigs from Trial#2 using the P0-2017 sequence as reference. Mutation location were indicated in nucleotide (nt) and amino acid (aa) sequences position, starting from the first nt of the 5′ nontranslated region (5′NTR) on the KF991509.2 full-length sequence and the start codon of each open reading frame (ORF), respectively.
